# Rabies in Morocco: Epidemiological Aspects and Post-exposure Prophylaxis Management

**DOI:** 10.7759/cureus.43658

**Published:** 2023-08-17

**Authors:** Kenza El Bazi, Touria El Bardi, Mouhcine Miloudi, Said Zouhair, Lamiae Arsalane, Youssef El Kamouni

**Affiliations:** 1 Microbiology, Avicenne Military Hospital, Marrakech, MAR

**Keywords:** immunoglobulin e, biting animal, vaccination, delay, post exposure prophylaxis, rabies

## Abstract

Background

Rabies is a zoonosis transmitted accidentally to humans and is fatal once clinical signs appear. In Morocco, rabies remains a difficult disease to eradicate, with dogs being the main source of contamination. Transmission of this illness can be avoided by promptly implementing post-exposure prophylaxis (PEP) following animal exposure. Inappropriate or delayed PEP increases the risk of acute infection. This study aims to describe and analyze the characteristics of exposure, investigate the factors related to delay in initiating PEP, and evaluate the attitudes and practices of patients towards animal exposure in the region of Ouarzazate between 2016 and 2019.

Methods

This was a retrospective study with statistical analysis. The data on animal exposure was collected from the Anti-Rabies Center (ARC) located in Ouarzazate Province, Morocco.

Results

A predominance of male cases (61.3%) among victims of animal exposure was found. The age group of five to 14 years was the most affected (23.5%). The upper limb represented the most frequent site of exposure (49.4%). Around 52.1% of patients presented with multiple lesions. About 54.4% of cases were bitten by a stray animal. Dogs were responsible for 47.8% of the bites. All exposed individuals performed wound cleansing prior to arrival at the ARC. Post-exposure prophylaxis was initiated in 34.5% of cases between 24 to 48 hours, while 26.1% of cases delayed it beyond 48 hours. Around 34% of patients did not complete their vaccination schedule. Regarding the factors associated with the delay in PEP, we found significant associations with patient age, place of residence (urban or rural), distance from the ARC (>30 km or <30 km), nature, number, and injury status (p≤0.05). Significant associations (p≤0.05) were also found with dog bites and their type (domestic or stray).

Conclusion

Our findings indicate that better awareness about rabies and PEP management is needed, especially among the rural population.

## Introduction

Rabies is a zoonosis transmitted accidentally to humans and is fatal once clinical symptoms appear. Fortunately, the transmission of this illness can be avoided when post-exposure prophylaxis (PEP) is promptly implemented following animal exposure. The WHO (World Health Organization) defines exposure to suspected or confirmed rabid animals in three categories (categories I, II, and III). Category I consists of touching or feeding animals, licking on intact skin, or contact on intact skin with the secretions of a rabid animal. Category II consists of nibbling of uncovered skin and minor scratches or abrasions without bleeding, and category III consists of transdermal bites or scratches, licks on broken skin, contamination of the mucus membrane with saliva, and exposure to bats. According to the WHO guidelines, PEP with immediate wound washing and vaccination is recommended for category II injuries, with the addition of rabies immunoglobulin (RIG) for category III injuries [1].

Despite the existence of effective vaccines for human and veterinary use, rabies remains a major public health problem, causing 59,000 deaths worldwide each year. Most cases occur in Africa and Asia; about 40% are children aged <15 years [1]. The etiological agents are grouped within the genus Lyssavirus. Rabies results in meningoencephalitis with a fatal outcome. There is no effective treatment for rabies once clinical signs appear. The therapeutic strategy is to eliminate the virus before it enters the nervous system. Inappropriate or delayed PEP increases the risk of acute infection [1].

Rabies is endemic in Morocco. Dogs are the main source of contamination. Every year, 21 cases of human rabies are biologically confirmed at the Pasteur Institute in Morocco [2]. Rabies continues to be a public health problem in Morocco despite the control actions implemented for many years by the rabies control program, deployed in 1986 by both the Ministries of the Interior and Health [3]. In Ouarzazate, according to the provincial epidemiology unit, the last case of human rabies was declared in 2001. However, the risk is present, with an average of 420 bites each year in the province [4].

Our work consists of an analytical retrospective study aiming to assess the characteristics of exposure, investigate the factors related to delay in initiating PEP, and evaluate the attitudes and practices of patients towards animal exposure.

## Materials and methods

Study zone

The province of Ouarzazate extends over an area of ​​12,464 km², i.e., 14% of the entire regional territory, and is subdivided into two urban and 15 rural communes [5]. The legal population of the province reached 297,502 inhabitants in 2014, according to the General Population and Housing Census 2014 (RGPH 2014) [5]. Per the 2014 census, the share of the rural population of the province (61.8%) greatly exceeds the urban population (38.2%) [5].)

Data sources

Data was collected in February 2021 from patient registers available at the Municipal Office of Hygiene of Ouarzazate. It is a service responsible for applying legal and regulatory provisions relating to public health and safety. Files of patients exposed between 2016 and 2019 were included while maintaining patient anonymity. Patients who were not from Ouarzazate Province were excluded from the analysis. 

A checklist was used to collect data from available registers at the Municipal Office of Hygiene of Ouarzazate. We collected information about epidemiological characteristics such as sex, age, place of residence (rural or urban), type of biting animal (dog or another animal, domestic or stray animal), and distance from the rabies treatment center. Details on clinical characteristics were also gathered, such as bite site (upper limb, lower limb, head, trunk, genital organs), and injury status (deep, superficial). Subjects exposed to animal bites who previously received treatment were cared for either at the Anti-Rabies Center (ARC) of Ouarzazate or at the Taznakhte Health Center, a communal health center (Commune of Taznakhte) located in the province of Ouarzazate. 

Socio-economic data, as well as related data such as population density, and housing type (urban or rural), were obtained from RGPH 2014. This study received the approval of the Comité d'éthique et de recherche-Hôpital Militaire Avicenne Marrakech (approval no. 4/2021).

Data analysis

Statistical analysis was performed using SPSS Statistics version 20.0 (IBM Corp., Armonk, NY, USA). This analysis is split into two parts: a descriptive analysis (numbers and percentages) and an analytical (univariate analysis by a binary logistic regression), using the chi-square test and Fisher's exact test for the comparison of frequencies. The significance threshold was set at 5% (p≤0.05)

## Results

Socio-demographic data

**Figure 1:**
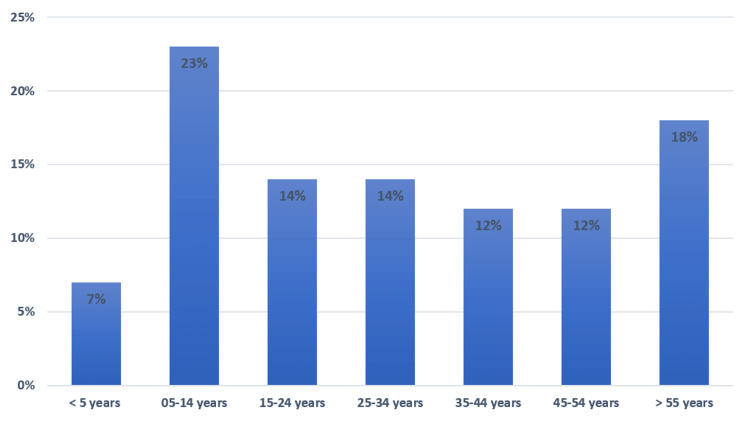
Animal exposure according to age groups

In total, 1758 cases of bites were recorded in the Ouarzate region between 2016 and 2019. Males were predominant, with 61.3% of exposed cases (n=1076). The M/F sex ratio was 1.58. This same predominance was observed in each year of the study. A predominance of animal bites in rural areas was noted (64.8%, n=1139). The average age of exposed cases was 31.12 years +/- 21.55. The median age was 28 years (11 to 48 years), with extremes ranging from seven months to 99 years. The age group of five to 14 years was the most affected with 23.5% of all cases (Figure 1). 

Characteristics of exposure

Upon arrival at the medical center, patients were clinically examined for signs of hemodynamic instability. All patients were noted to be hemodynamically stable, and so was their breathing. An examination of the wounds in the event of a bite was then initiated.

The most frequent site of exposure was the upper limb in 49.4% of cases, followed by the lower limb in 39.8% of cases. Head injury came in third with 5.2% of cases, followed by trunk injury, combined involvement of the upper and lower limbs, and genital organs with 4.6%, 0.9%, and 0.1%, respectively.

Regarding the nature of exposure, bites accounted for 76.9%, scratches for 17.9%, and contact-type exposure for 5.2%. Exposure did not cause any lesions in 5.2% of patients (licking or contact with drool). Injuries caused were superficial in 62.6% of cases and deep in 32.2%. Around 52.1% of the cases presented multiple lesions, and 42.7% presented single lesions.

Medical care following animal exposure (wound treatment, tetanus prophylaxis, rabies prophylaxis)

A total of 1758 people were cared for at the Ouarzazate ARC. The median time between exposure and PEP was 1 day (0 to 2 days). Post-exposure prophylaxis was initiated on the same day in 39.4% of cases and between 24 and 48 hours in 34.5% of cases. People who delayed their PEP beyond 48 hours accounted for 26.1% of cases. A downward trend in PEP delays beyond 48 hours was noted in 2017 with 31% of cases, 25% of cases in 2018, and 22% in 2019.

The cleaning of wounds in exposed cases was carried out systematically (100% of cases) with various products (hot water and soap, alcohol, bleach) before patients arrived at the ARC and with a povidone-iodine solution while at the Ouarzazate ARC. Of the 1758 cases in our series, seven presented serious wounds requiring stitches. Antibiotic prophylaxis was indicated for high-risk wounds (hand bites, massive crush injuries, immunocompromised patients) and was only prescribed in 61 patients (3.5%). Concerning tetanus prophylaxis, only 11.4% of patients received anti-tetanus immunoglobulin (TIG), while the remaining 88.6% did not require it. 

**Figure 2:**
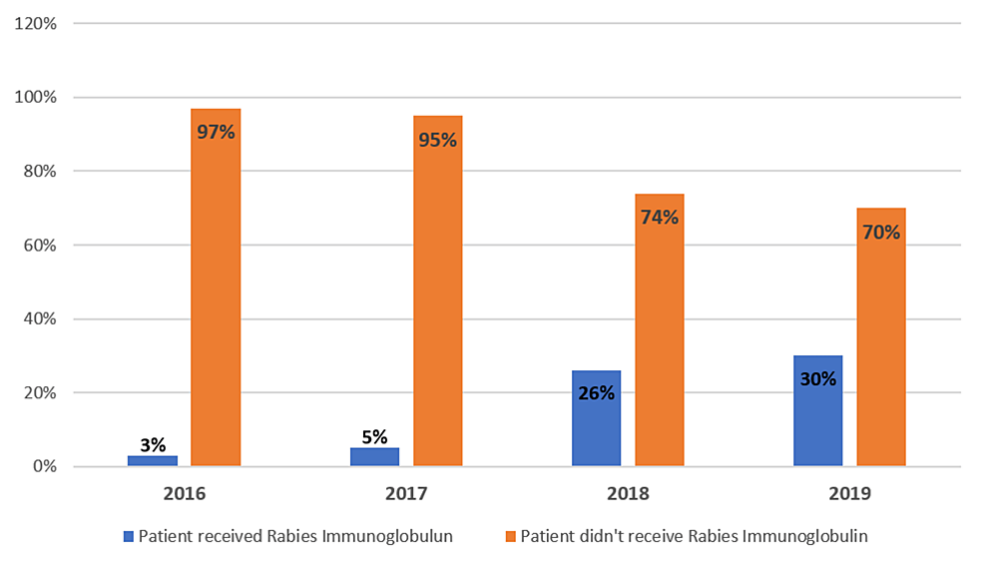
Rabies immunoglobulin administration between 2016 and 2019

Around 83.3% of exposed cases did not receive rabies immunoglobulin (RIG). An increase in the administration of RIG was noted over the years (Figure 2). Concerning rabies vaccination, only 68% of patients received a complete rabies vaccine schedule with a 2-1-1 regimen. No adverse reactions were reported.

Type of animal bites 

Dogs were responsible for 47.8% of exposures. Cats came in second with 33.6% of reported cases, and mules placed third with 15.3% of reported cases. About 54.4% of patients were attacked by stray animals. The diagnosis of rabies in animals was suspected clinically in 97.4% of cases when the animal could be captured and was confirmed by molecular biology in 1.1% of cases.

Factors influencing delay in initiating PEP

Different factors influenced the delay of PEP. Table 1 below shows the results found.

**Table 1:** Associated factors with delay in initiating PEP PEP: Post-exposure prophylaxis, ARC: Anti-rabies center, OR: Odds ratio, CI: Confidence interval

Variables	Characteristics	Delay in PEP		Statistical analysis		
		>48H	≥48H	OR	CI	p-value
Age	≤15 years	432	129	-	-	-
	>15 years	859	329	1.2	1.015-1.6	0.037
Residence	Urban	497	124	-	-	-
	Rural	800	334	1.6	1.32-2.1	<0.001
Distance from the ARC	<30 km	778	203	-	-	-
	>30 km	520	255	1.8	1.5-2.3	<0.001
Nature of injury	Category III	1018	331	-	-	-
	Category II	238	75	0.96	0.72-1.29	0.83
	Category I	39	52	4.1	2.65-6.32	<0.001
Number of lesions	Multiple	707	206	-	-	-
	Unique	548	201	1.25	1-1.57	0.044
	Contact	39	51	4.48	2.87-7	<0.001
Injury status	Deep	443	122	-	-	-
	Superficial	813	285	1.27	1-1.62	0.05
	Contact	39	51	4.74	2.9-7.5	<0.001
Biting animal	Dog	643	192	-	-	-
	Cat	442	146	1	0.8-1.4	0.46
	Mule	167	101	2	1.49-2.69	<0.001
	Rat	17	3	0.5	0.17-2.03	0.4
	Horse	5	5	3.3	0.95-11.6	0.058
	Camel	5	1	0.6	0.07-5.76	0.71
	Fox	5	2	1.34	0.25-6.95	0.72
	Monkey	2	1	3.35	0.2-53.7	0.39
	Wolf	1	1	3.35	0.2-53.7	0.39
	Squirrel	0	1	-	-	-
	Pig	1	0	-	-	-
Type of biting animal	Domestic animal	726	223	-	-	-
	Stray animal	567	230	1.3	1.06	0.01

Patient follow-up after receiving PEP

All of the patients in the current study were healthy and alive one year after receiving PEP. We have not observed any case of rabies in any of the patients who have received PEP. In Morocco, the reporting of human rabies cases is mandatory.

## Discussion

Exposure to animal bites is a public health problem given the risk of human rabies. In the province of Ouarzazate, the last case of human rabies dates back to 2001. An average of 20 cases is still declared annually in our country [3]. The average exposure rate to animal bites in the province of Ouarzazate was estimated at 0.19% or 190 per 100,000 inhabitants. Another national study carried out in the province of Sidi Kacem in 2008 estimated the exposure rate at 0.21% (210/100,000 inhabitants) [6]. The following table presents a comparison of different exposure rates per 100,000 inhabitants based on national [6] and international [7-10] data (Table 2).

**Table 2:** Exposure rates to bites in the literature

	Morocco, Sidi Kassem (2008) [6]	Iran (2011) [7]	Tunisia (2004-2018) [8]	Brazil (2008-2017) [9]	Iran (2015-2017) [10]	Our study, Morocco, Ourzazate (2016-2019)
Average number of cases per year of study	687	7097	3089	506	724	440
Exposure rate (per 100,000 inhabitants)	210	154	694	255	242	190

Different socio-demographic elements have been found to influence the risk of exposure. Age seems to be a predisposing factor to bites; the youngest subjects were the most exposed, with 32.1% of cases reported in the population under 15 years old. African literature in Côte d'Ivoire shows the same trends [11]. This can be explained by the lack of vigilance among the youngest. Despite this predisposition to bites among that age group, the older population is more likely to delay PEP beyond 48 hours. We did not find similar findings in the literature; however, a Tunisian study [8] found an association between belonging to a certain category of the population (students, housewives, and farmers) and the delay of PEP. This finding could partly explain our result. The population over 15 years old is generally active and lacks time because of work or studies.

A predominance of animal bite cases in rural areas was noted (64.8%). Literature shows different findings, with 87% of cases reported by a previous study in urban areas [11]. Our results showed that people living in rural areas were more likely to delay their PEP, and the same observation is found in the literature [12,13]. In 44% of cases, the nearest ARC was more than 30 kilometers away. A statistically significant relationship was found between PEP delays and distance from ARC. Other studies have found similar results [14]. In our study, dogs were implicated in 47.8% of cases of exposure, a finding that has been found in the literature [15]. Patients bitten by an animal other than a dog tended to delay PEP. This can be explained by the erroneous perception of the population about the category of animals likely to transmit the disease. Cases bitten by stray animals accounted for 54.5% of cases. Gebrü et al. [15] also found a predominance of this category of biting animals (80%). Victims bitten by an animal with an owner are late in reporting to the ARC. These results are in agreement with those of a previous study [16]. Category III injuries represented 76.8% of exposures. Similar results were found in the literature [12]. We found that the low number, as well as the superficial nature of the wounds, is a factor associated with the delay of PEP. The same finding is backed by the literature [12].

Post-exposure prophylaxis mainly consists of a curative vaccination that is mandatory in the event of suspected contamination. The vaccination protocol includes four intramuscular injections (protocol D0:2/ D7:1/ D21:1). Vaccination can be combined in cases of serious contamination with anti-rabies serotherapy [3]. The human rabies vaccine currently marketed in Morocco is the inactivated rabies vaccine VERORAB (Aventis Pasteur), distributed by the Pasteur Institute of Morocco [2]. Our findings show that most of the bitten patients (89%) received the first two doses of the vaccine, and 76% of patients completed the vaccination schedule. The rates found remain higher than those of other countries [17,18].

Finally, the fact that data was collected from the ARCs at Ourzazate and Taznakhte can be considered a limitation of this study since it can lead to missing out on a majority of patients living in remote areas who were not able to access these centers for care. We could only evaluate the delay in PEP, but another study evaluating the absence of PEP for patients living in rural areas is needed to clarify this issue. 

## Conclusions

Despite a decline in confirmed cases of human rabies in Morocco, data collected in the region of Ouarzazate demonstrate that exposure to animal bites is still raging in Morocco. Clinical examination of patients showed that the most frequent sites of exposure were the upper limb and the lower limb. Regarding the nature of exposure, bites were more frequent, followed by scratches and contact-type exposure. We observed a delay beyond 24 hours in the initiation of PEP in more than a third of the population studied. Factors associated with delayed PEP were rural location, non-severe bite wounds or contact-type lesions, and being bitten by an animal other than a dog. Our results demonstrate that PEP delays are generally due to socio-demographic factors. The key to a PEP carried out as soon as possible is awareness, especially in rural and remote areas, as well as the accessibility of ARCs. This clearly demonstrates the need for health education programs, principally targeting rural areas.
